# Climate Degradation and Extreme Icing Events Constrain Life in Cold-Adapted Mammals

**DOI:** 10.1038/s41598-018-19416-9

**Published:** 2018-01-18

**Authors:** J. Berger, C. Hartway, A. Gruzdev, M. Johnson

**Affiliations:** 1 Fish, Wildlife and Conservation Biology, Colorado State University, Fort Collins, CO 80523 USA; 2 Wildlife Conservation Society, Bronx, NY 10460 USA; 3 Department of Natural Resources, South Dakota State University, Brookings, SD 57007 USA; 4 Wrangel Island World Heritage Site, Pevek, Chukotka Autonomous Zone, Russia; 5 US Forest Service, Tongass National Forest, Juneau, AK 99801 USA

## Abstract

Despite the growth in knowledge about the effects of a warming Arctic on its cold-adapted species, the mechanisms by which these changes affect animal populations remain poorly understood. Increasing temperatures, declining sea ice and altered wind and precipitation patterns all may affect the fitness and abundance of species through multiple direct and indirect pathways. Here we demonstrate previously unknown effects of rain-on-snow (ROS) events, winter precipitation, and ice tidal surges on the Arctic’s largest land mammal. Using novel field data across seven years and three Alaskan and Russian sites, we show arrested skeletal growth in juvenile muskoxen resulting from unusually dry winter conditions and gestational ROS events, with the inhibitory effects on growth from ROS events lasting up to three years post-partum. Further, we describe the simultaneous entombment of 52 muskoxen in ice during a Chukchi Sea winter tsunami (*ivuniq* in Iñupiat), and link rapid freezing to entrapment of Arctic whales and otters. Our results illustrate how once unusual, but increasingly frequent Arctic weather events affect some cold-adapted mammals, and suggest that an understanding of species responses to a changing Arctic can be enhanced by coalescing groundwork, rare events, and insights from local people.

## Introduction

In August 2015, President Barack Obama visited the small town of Kotzebue in Arctic Alaska to garner an increased understanding of the immediacy and potential consequences of warming global temperatures. On a day in February that year, the low temperature in Kotzebue failed to drop below 0 °C for the first time; the snow melting as it rained. Fifteen hundred kilometers northeast and thirteen years before, a major winter rain-on-snow (ROS) event encased the ground in ice preventing the Arctic’s largest land mammal from accessing food; an estimated 20,000 muskoxen (*Ovibus moschatus*) died^1^. Nearly 150 years earlier, Charles Darwin noted the primacy of abiotic conditions in determining the fitness of species at the top of the world by observing: “Only in extreme conditions in arctic regions or on the borders of an utter desert will competition cease and the struggle for life become almost exclusively with the elements”^2^.

The Arctic is a realm of extremes, and Arctic species have adapted to maximize short growing seasons and endure long winters that may include temperatures below −40 °C, driving winds, and deep snow. A changing climate now brings new challenges for arctic species; warming temperatures are altering air velocity and the extent and duration of sea ice, all of which conflate to modify ocean currents and the timing and intensity of precipitation^3^. As a result, once rare phenomena such as ROS, drought, and tidal surges are increasing in frequency^4^. Yet the effect of these phenomena on the fitness and abundance of Arctic wildlife remain little understood, as research efforts are hindered by high latitude remoteness and complex logistics^5,6^. For example, research on effects of Arctic drought and tidal surges are concentrated almost exclusively on plant and freshwater communities^7–9^ whereas study of ROS has focused primarily on extreme events (>10 mm) owing to the catastrophic effects of such events on wildlife^1,10,11^. In the arid regions of Beringia, extreme ROS events are rare, yet the frequency of minor events (<10 mm) is increasing^12^. While not likely to be as catastrophic to wildlife as extreme ROS, smaller events may still lead to partial or complete ground icing^11^ and less access to food, with potentially negative effects on fitness.

Assessing the effect of weather variance associated with warmer temperatures on herbivorous Arctic species is further complicated by the myriad direct and indirect pathways by which weather can affect the energy balance of herbivores^13^. For example, warmer winter temperatures coupled with precipitation can have indirect negative effects on herbivores by increasing the probability of ROS ground-icing events which limit access to food resources^11,14^. Yet warmer winter temperatures may also have a positive effect on the energy balance of an individual by ameliorating thermoregulatory costs. Likewise winter precipitation falling as rain can reduce snowpack, thereby reducing the metabolic costs of foraging in deep snow^15,16^. However snowpack also insulates ground vegetation^17,18^, and thus reduced snowpack could negatively affect plant productivity and resource availability in the following growing season.

**Figure 1:**
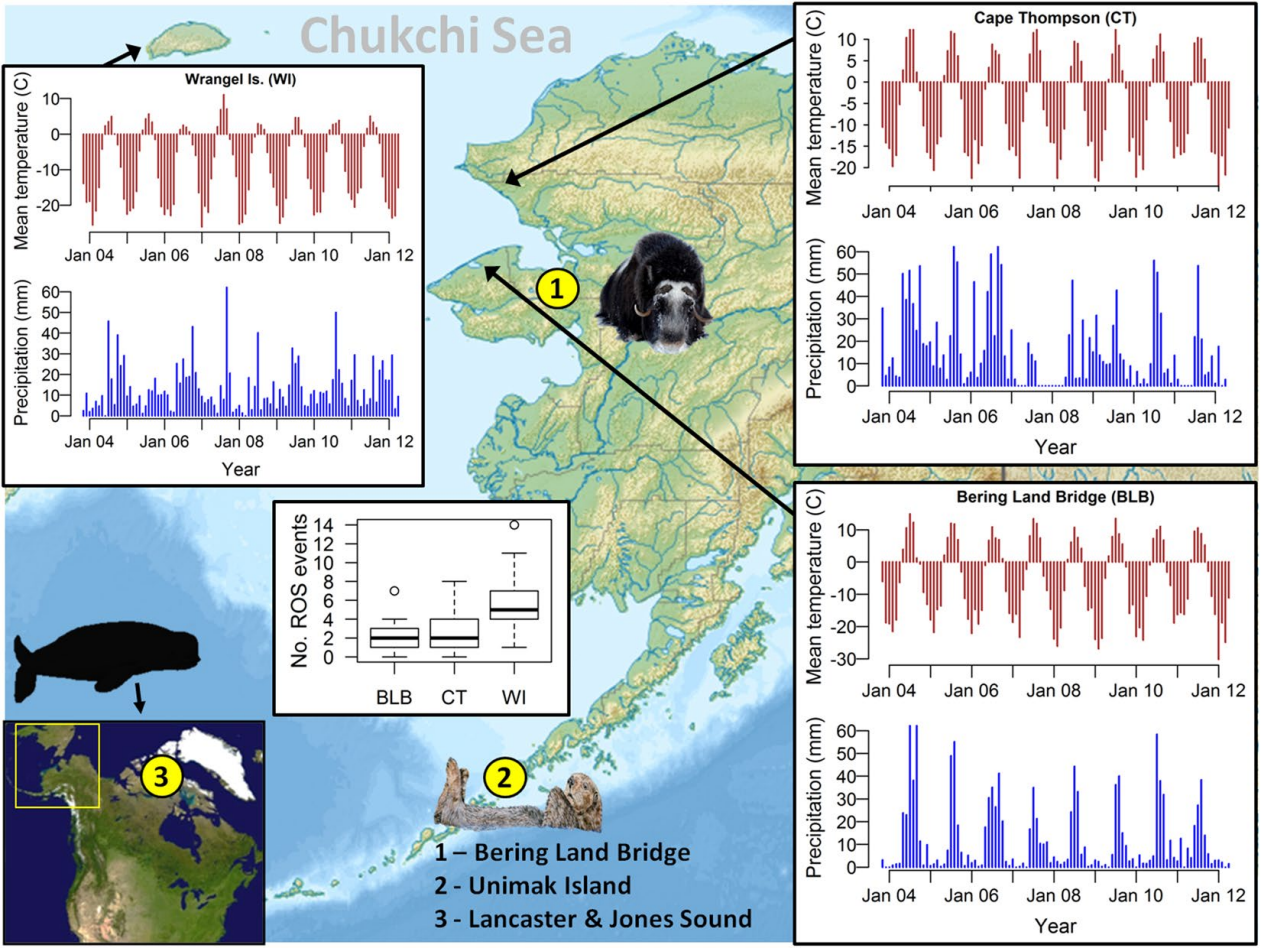
Monthly weather profiles (2004–2012) and number of ROS events/year (2006–2012) for our three study sites: Bering land Bridge (BLB), Cape Thompson (CT), and Wrangel Island (WI). Yellow circles and figurines denote locations and species/taxa affected by extreme icing events discussed in main text. Map of Beringia was modified by the authors from the original by Nikita A. Zemin (https://en.wikipedia.org/wiki/Juneau,_Alaska#/media/File:Relief_map_of_USA_Alaska.png), CC-BY SA 3.0 (https://creativecommons.org/licenses/by-sa/3.0/).

**Figure 2:**
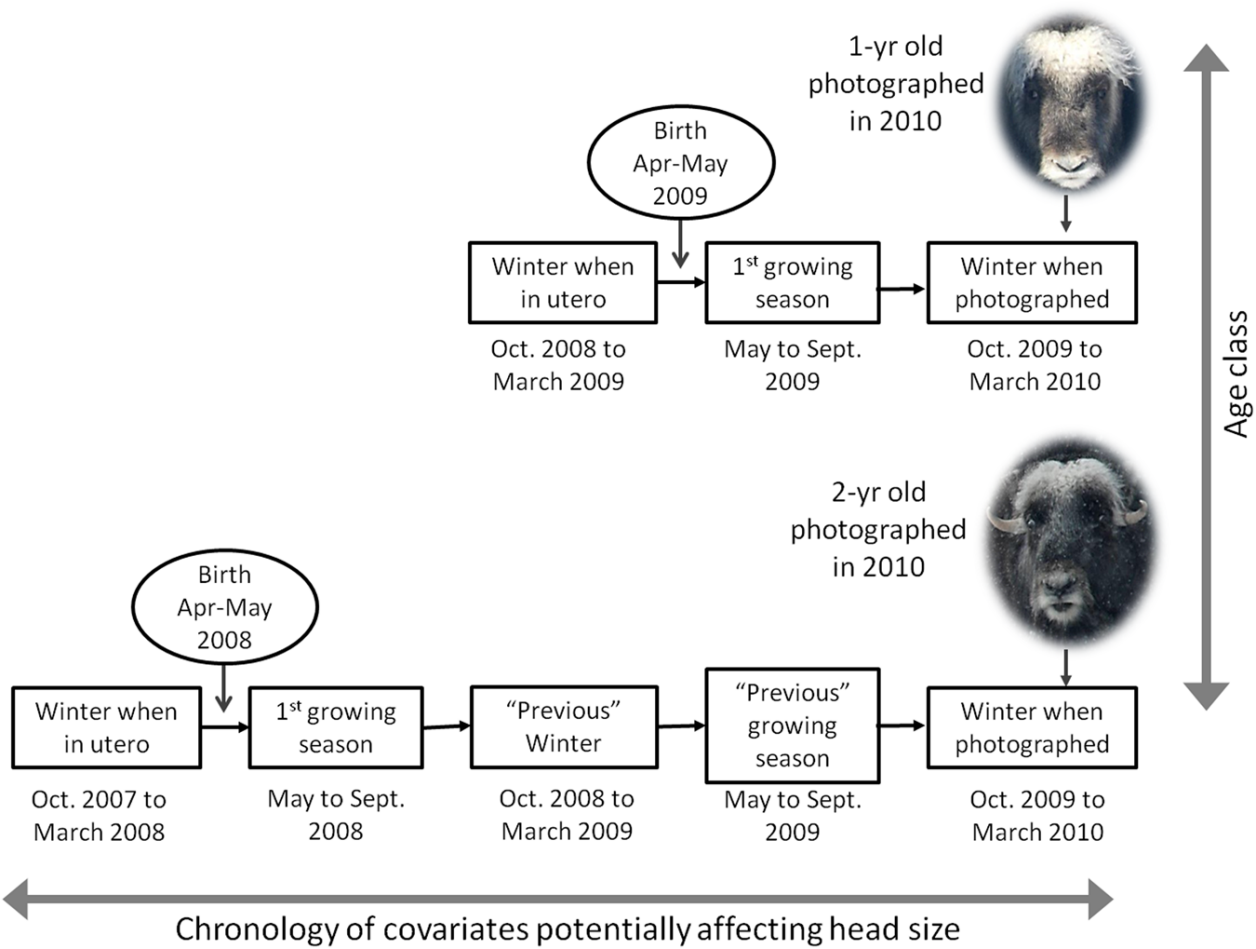
Schematic showing the timing of covariate data relative to when animals were photographed for: (top) 1-yr old; and (bottom) 2-yr old muskoxen (3-yr olds not shown). Path analysis models for each age class included winter condition covariates (extreme cold events, ROS events and mean precipitation) from the “previous winter” and from the winter when an individual was in utero; and growing season related covariates (iNDVI) from the “previous growing season” and for an individual’s “first growing season”. For 1-yr old muskoxen, the previous winter and previous growing season are the same as “in utero” and “first growing season”, respectively.

**Figure 3:**
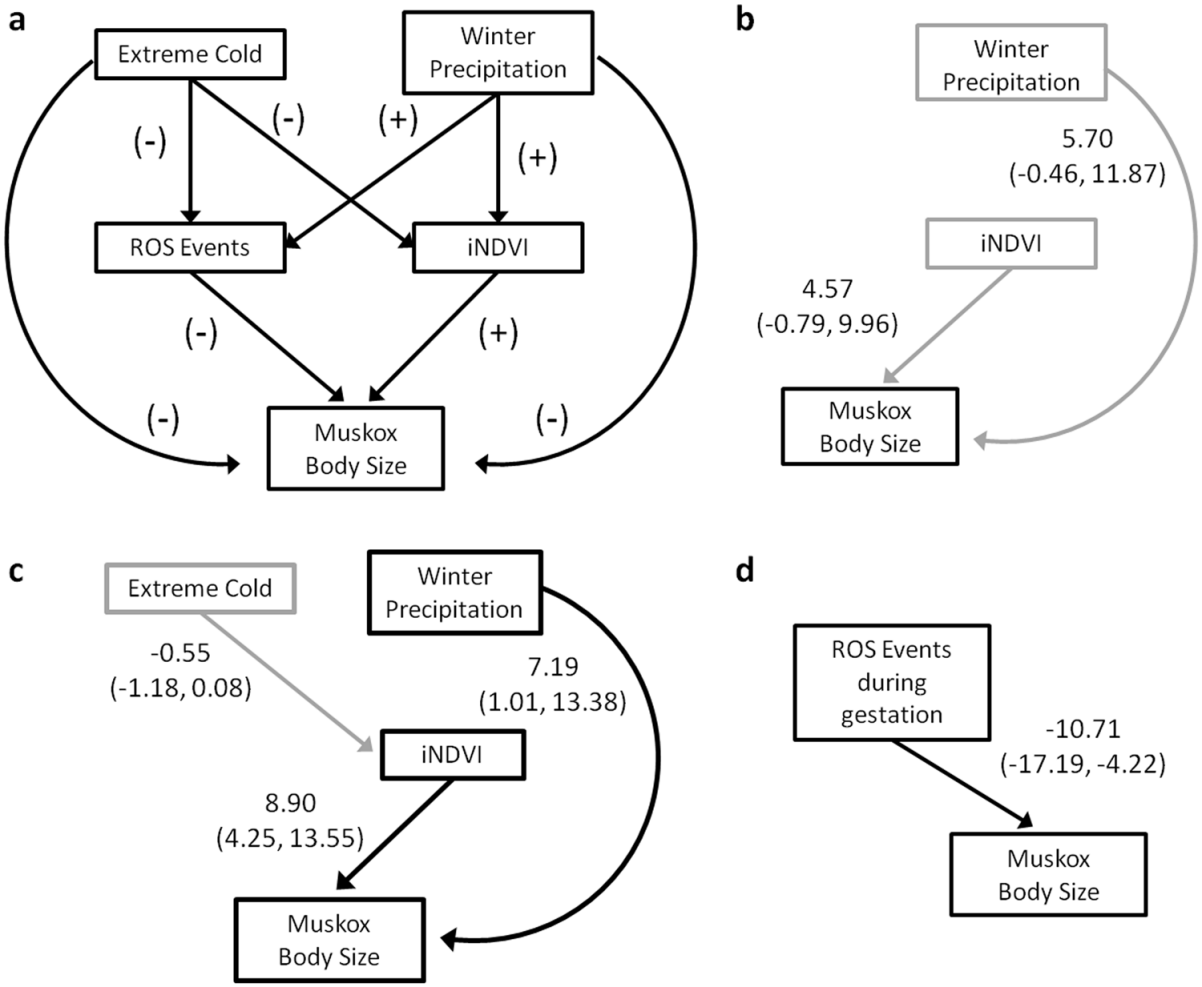
Initial conceptual model (**a**) of the potential direct and indirect effects of weather and food resources on muskoxen body size; and best models as determined by model selection for (**b**) 1-yr old; (**c**) 2-yr old; and (**d**) 3-yr old muskoxen. Mean posterior values and 95% credible intervals (in parenthesis) are given for standardized coefficients of each effect. Only results from links that directly or indirectly affect muskoxen are shown. Dark lines indicate 95% CI do not include 0, light lines indicate marginal inclusion of 0.

In this study we present data and observations on the direct and indirect effects of unusual and extreme weather variance on the skeletal growth and survival of muskoxen. We demonstrate effects of weather variance on the skeletal growth of muskoxen by analyzing head size dimensionality from digital photo data (see Methods) from 1-, 2-, and 3-year old individuals across six years at two sites in Arctic Alaska (Fig. 1). We first use Bayesian path analyses - a type of multivariate analysis that can account for multiple, casual relationships between covariates^19^ - to model head size as a function of extreme cold, mean winter precipitation, number of ROS events and growing season plant productivity (Table 1). Because juvenile skeletal size is the cumulative product of nutritional conditions and metabolic costs throughout an animal’s life^20–28^, we included in our models individual-specific covariate values recorded during the growing season and winter previous to when the individual was photographed, the winter when the individual was in utero, and during its first growing season (Fig. 2). Our conceptual model of potential relationships between covariates of interest, and between covariates and muskoxen head size is given in Fig. 3a. To further assess the effects of spatial variation in environmental conditions, we compare whether the mean head size of 1-, 2-, and 3-yr olds from a population in Wrangel Island – a colder site in the Asian Arctic that is more prone to minor ROS events – were within the 90% confidence interval for the Alaskan animals.

**Table 1:** Variables of interest for modeling the effect of weather and food resources on muskoxen juvenile growth, the covariates and data used in models to approximate these variables, and the range of each.

Variables of interest	Observed data used in models	Variable range
Winter snow depth	Mean of monthly precipitation in winter (Nov. to April). Calculated from monthly weather station data.	0 to 16.9 mm
Extreme cold events	Number of days when the temperature dropped below −23 °C (Nov. to April). Calculated from daily weather station data.	8 to 47 events
Rain-on-snow events	Number of days (Oct. to May) when precipitation was >0 mm, temperature was >0 °C and snow was presumed to be on the ground (e.g. a precipitation event with temperature <0 °C had previously occurred). Calculated from daily weather station data.	0 to 8 events
Resource availability	Standardized time integrated NDVI (iNDVI) averaged over all polygons at each study site.	−2.4 to 1.6
Body Size	Head size dimensionality as estimated by photogrammetry.	138.0 to 280.0 cm2 (1yo) 214.0 to 337.6 cm^2^ (2yo) 263.9 to 387.0 cm^2^ (3yo)

To illustrate the effects of extreme weather variance on muskoxen survival, we present observations of a mass mortality event caused by a singular wind-driven ocean tidal surge. Such offshore events are known to affect terrestrial species, yet documentation beyond meteorologically-driven storm paths leading to food deprivation by deep snow or icing remains uncommon. Finally, we supplement the generality of our findings by linking field observations with traditional ecological knowledge to further illustrate how extreme icing impacts other Arctic species.

## Results

Results of our path analysis suggest that mean head size of 1- and 2-yr old muskoxen is positively associated with increased precipitation in the winter, and with plant productivity during the growing season (iNDVI) (Fig. 3b,c). Results also suggest an indirect, negative effect of extreme cold on the mean head size of 2-yr olds mediated through an inhibitory effect on plant productivity during the growing season (Fig. 3c). In contrast, variation in the head size of 3-yr old muskoxen was only associated with variation in the frequency of minor ROS events during gestation (Fig. 3d). The mean head size of 3-yr olds decreased with an increase in the number of minor ROS events occurring while animals were in utero.

**Figure 4:**
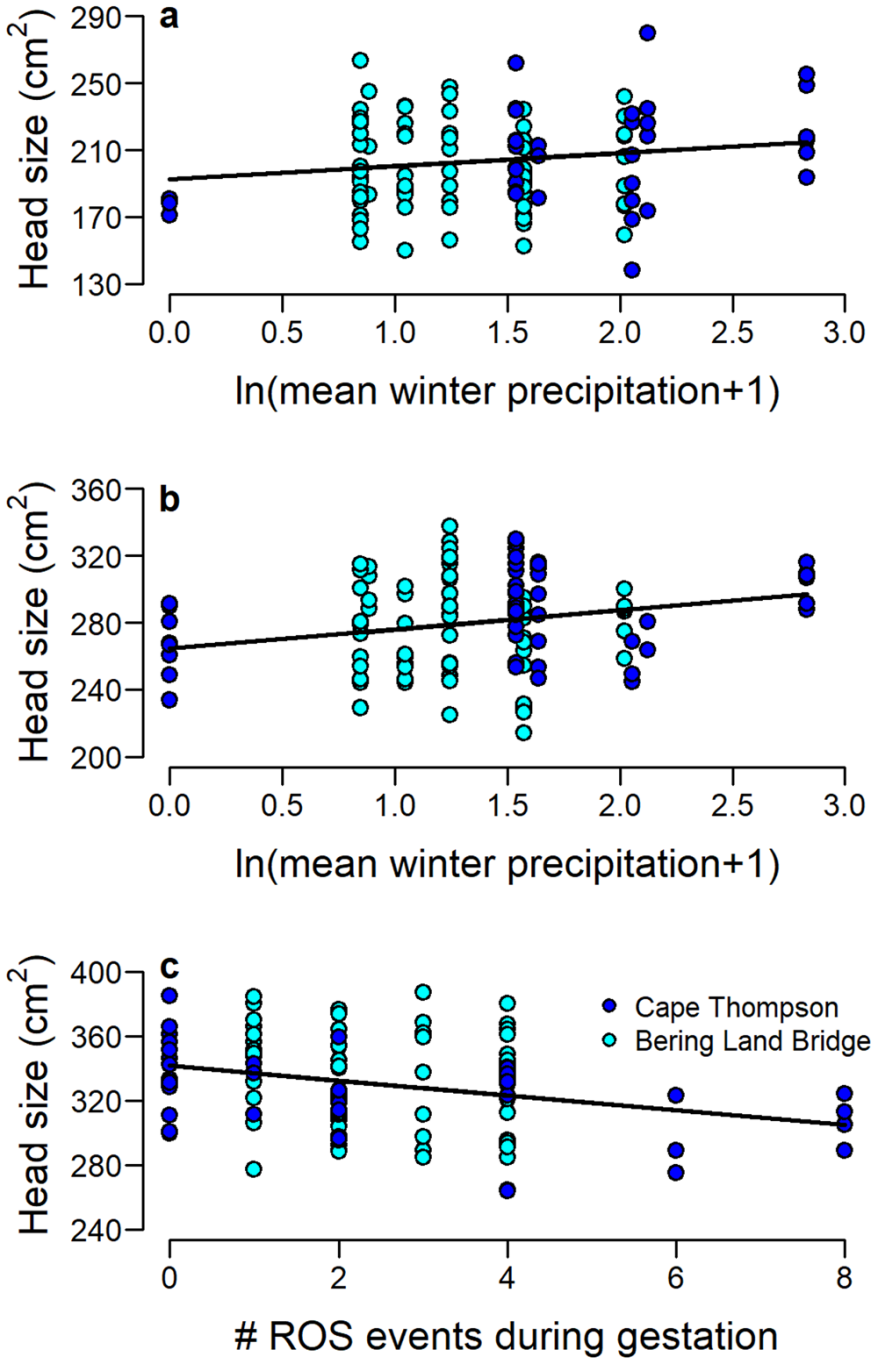
Relationships between head size and winter precipitation (with iNDVI held constant) for (**a**) 1-yr old; (**b**) 2-yr old; and (**c**) gestational ROS events for 3-yr old muskoxen. Lines indicate predicted values based on parameter estimates from Bayesian analyses.

The relationship between mean head size and precipitation for 1- and 2-yr olds was driven by the extremes in our dataset (Fig. 4a,b). On average, the smallest animals in the 1- and 2-yr old age classes were observed after the winter of 2007–2008 in which no precipitation was recorded between the months of October and April (Fig. 1; −100% deviation from normal – see Methods), and the largest after the wettest winter in our dataset (2008–2009: monthly mean of 16 mm, 10% above average). For 3-yr olds, the largest individuals, on average, were those that experienced no ROS events during gestation; while the smallest 3-yr olds experienced ≥6 (Fig. 4c). Best models for 1-, 2- and 3-yr olds predicted 18%, 13% and 23% of variation in head size, respectively, for Cape Thompson animals; but only 0%, 16% and 1% of variation for animals at Bering Land Bridge, suggesting that most of our results were driven by data from the Cape Thompson population.

**Figure 5:**
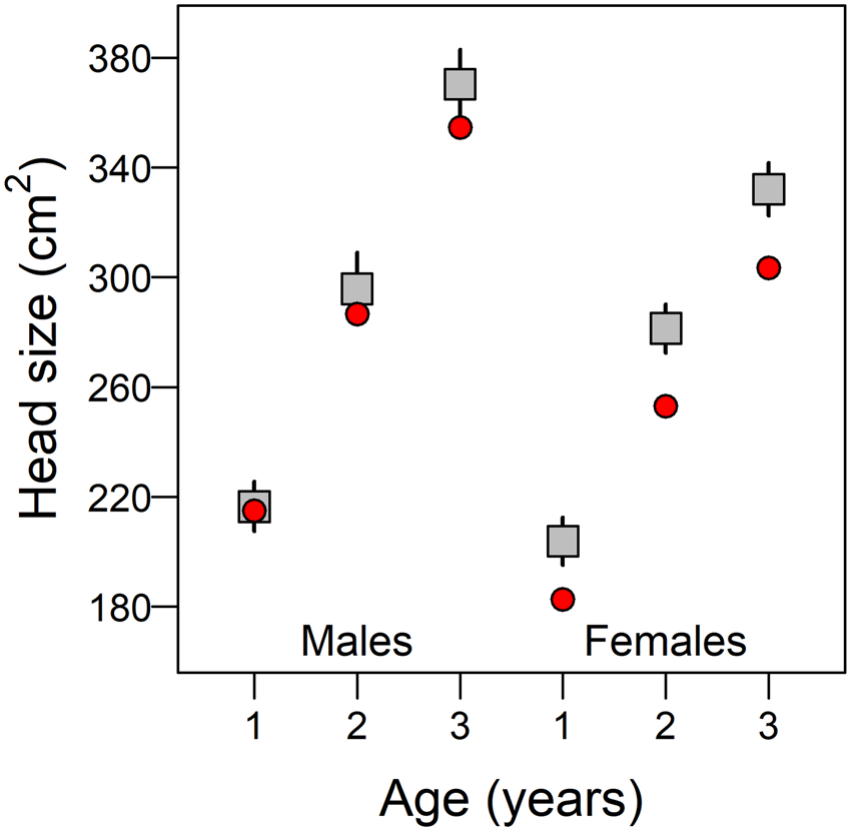
Mean head size and 99% confidence intervals (CI) by sex and age cohort for Alaskan data gathered between 2008 and 2013 (squares; N = 628), and mean head size data for Wrangel Island gathered in 2014 (circles; N = 45).

In the East Asian sector of Beringia on Wrangel Island, minor ROS events were more than 2× more frequent, temperatures colder and growing seasons shorter than at Alaskan sites (Fig. 1). Correspondingly, mean head size of Wrangel Island muskoxen were smaller than their Alaskan counterparts. The mean head size of 2- and 3-yr old males fell below the 90% confidence intervals, and the mean of each female cohort below 99.9% of their Alaskan counterparts (Fig. 5). The binomial probability of being a cumulative simultaneous outlier is p < 0.0001. Given that all muskoxen at our study sites are descended from the same Greenland founders (see Methods), our detection of such high variation in head dimensions suggests plasticity in size as a response to environmental variation^29^ although the extent to which Wrangel’s ROS, temperature and summer NDVI are conflated is unclear.

**Figure 6:**
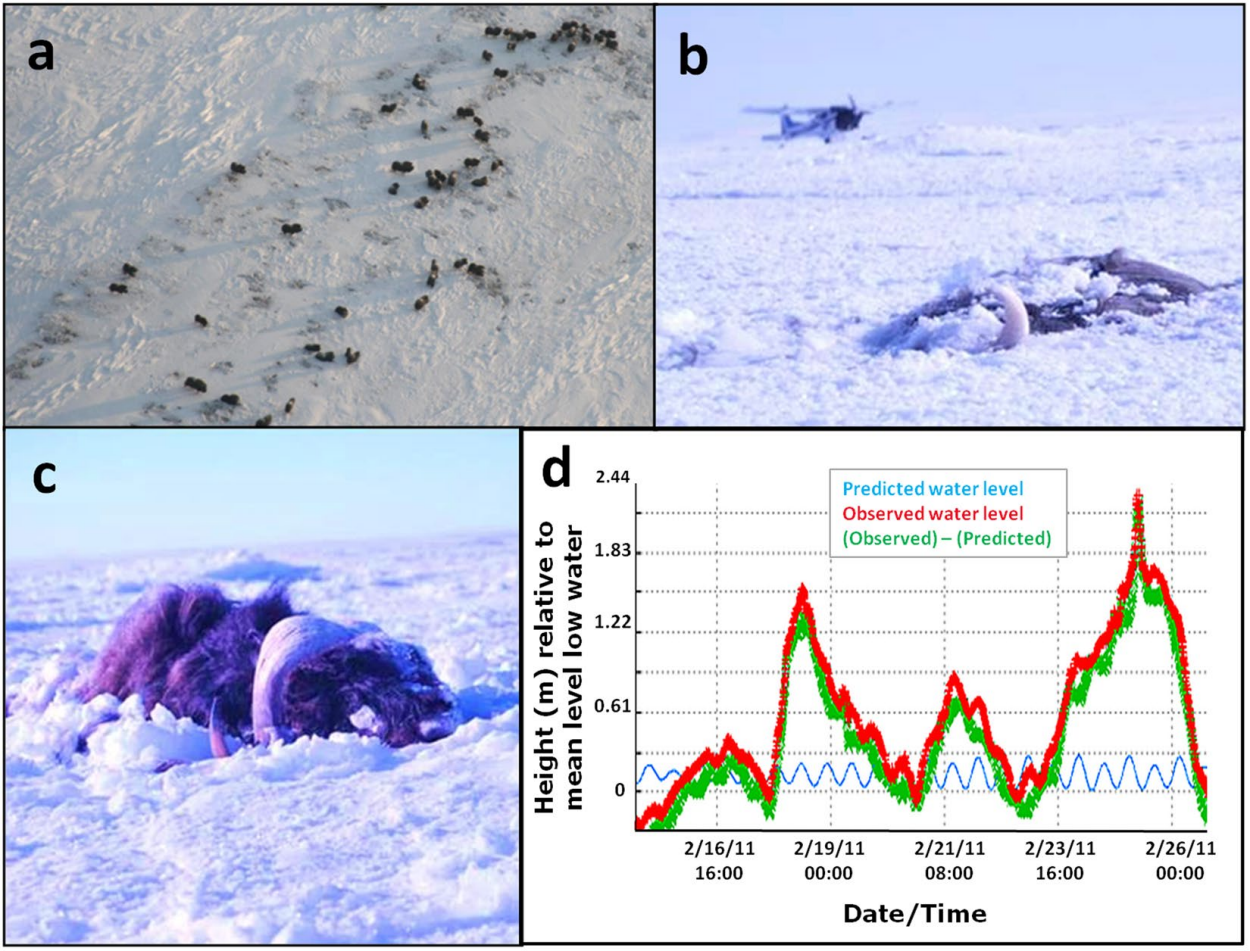
Overview of *ivu* mortality event. (**a**) Herd of muskoxen in Bering Land Bridge, Alaska on February 14, 2011; (**b**) and (**c**) the two most visible carcasses in March, 2011; (**d**) normal tidal flow at NOAA’s Red Dog station, along with predicted and observed water levels before and after the wind-whipped tidal surge (source: NOAA – see Methods).

In contrast to the subtle interactive effects detailed above, a one-time extreme icing event caused by a tidal surge produced a more certain effect – an extraordinary mass mortality event. On February 14, 2011, 55 muskoxen clustered in a lagoon at the northern coast of Bering Land Bridge peninsula (Figs 1 and 6a). A few days later, the closest NOAA-maintained monitoring station (the Red Dog Dock station – see Methods) registered a tidal episode with a historic high, 16× greater than normal (Fig. 6d). Accompanied by a fusion of shattered shore ice with plates up to 50 cm thick and 5 m long, the tidal surge trapped at least 52 animals. All but one was wholly submerged (Fig. 6). Known as an *ivu* or *ivuniq* in Iñupiaq, the language of indigenous northern-coastal Alaskans, these wind-driven polar ice-override surges pile ice to 4 m high, and distribute marine subsidies inland to 20 kms^30^.

## Discussion

Our findings on the inhibitory effects of exceedingly dry winter conditions and minor ROS events on the juvenile growth of muskoxen, and of mass ice–induced mortality due to a wind-driven tidal surge, offer potent new insights into the effect of unusual weather variance on the biology of cold-adapted species. Smaller-size in juveniles and sub-adults is typically associated with lower fitness due to delayed puberty and increased mortality^31–33^. However further longitudinal studies will be required to determine whether the negative effects of dry winter conditions and minor ROS events on juvenile growth ultimately affect the survival or reproduction of muskoxen, or whether compensatory growth occurs at later ages^34,35^.

The exceedingly dry winter in our dataset was part of a longer precipitation anomaly at Cape Thompson that began in April 2007 (Fig. 1), with total annual precipitation only 32% of the 20-year average. Further investigation with longitudinal data will be required to determine whether the negative effect of dry winter conditions on the growth of 1- and 2-yr old muskoxen stems from unfavorable conditions during winter, unfavorable effects on food resources during the growing season^13,20^, or both. However extremely dry conditions and lack of access to water in winter could become serious for some cold-adapted mammals under climate change. For instance, when snow patches (the only available winter water source) are scarce above 4500 m on the Tibetan Plateau, endangered wild yaks (*Bos mutus*) are less able to support lactation during winter^36^.

Why does the skeletal growth of 1- and 2-yr old muskoxen appear to be most negatively affected by exceedingly dry conditions, while the growth of 3-yr olds is most affected by ROS events during gestation? One potential explanation for this apparent discrepancy is the interdependence of ROS events and precipitation. In particular, the driest winter had no ROS events (0 events), meaning that for 1- and 2- yr olds the smallest head sizes were associated with the fewest ROS events. Our initial models suggested a weak negative association between gestational ROS events and head size for 1- and 2-yr old muskoxen, but these pathways were not retained during model selection. For these cohorts, it appears that negative effects associated with small increases in the number of ROS events were superseded by the positive effects of increasing precipitation.

In contrast, some of the smallest 3-yr olds in our dataset experienced both the driest winter as juveniles, and the most ROS events while still in utero (8 events). Our models identified ROS to be the stronger predictor of head size in 3-yr olds, but it is possible that both dry conditions and ROS events contributed to the slower growth of these individuals. A longer or more spatially extensive dataset - capturing greater variability in precipitation and ROS events -would allow for a more comprehensive evaluation of the relative strengths of these environmental conditions on each age class, and for the exploration of possible non-linear relationships between variables.

With the notable exception of polar bears and several other marine mammals, the acquisition of knowledge about the impact of a rapidly altering Arctic has progressed largely due to climate and habitat modeling bolstered by remote sensing advances rather than by ground knowledge of mammalian responses. Despite significant advances about the effects of icing on reindeer^11,37,38^, there is far more uncertainty than certainty about pattern and effect for a broader realm of species and taxa. Yet one-time events resulting from severe storms and changing ice conditions have been shown to affect species beyond the one-time iced entombment of 52 muskoxen (Fig. 6). For example, historical records include observations of spectacular sea ice entrapments of marine mammals, including approximately 150 narwhals (*Monodon monoceros*), 170 belugas (*Delphinapterus leucas*), and sea otters (*Enhydra lutris)* in southern Beringia, and also of a convergence of nine polar bears at a 20 × 6 m opening in sea ice, killing an estimated 100 belugas in the eastern Arctic^39,40^ (Fig. 1). While infrequently observed by scientists, these events carry the name *sassat* in Greenlandic, meaning “concentrated animals offered as food”. That the lexicon of Inuit culture^41^ includes the words *ivus* and *sassats* indicates that traditional ecological knowledge has long identified such critical climate-induced events, and such knowledge may offer important context for appreciating the frequency and impact of extreme weather variance on Arctic species^42,43^.

Our results add to a growing body of literature on the mechanisms by which climatic modification affects not only cold-adapted marine species but now also terrestrial ones. While modern technologies including satellite imagery, remote sensing, and climate modeling have improved our understanding of the changing Arctic environment, they cannot provide a complete substitute for knowledge derived by observation and fieldwork, comparative frameworks, and repositories of indigenous knowledge. Societal concerns about a changing Arctic include not just the loss of global biodiversity^4,5^, but also basic food security, and militarization. Subsequently, much is to be gained by coalescing on-the-ground approaches, reaching across increasingly tense geopolitical boundaries^44^, and incorporating knowledge of rare events and from local people.

## Methods

### Study species

Muskoxen were extirpated from Alaska within 20–25 years after its’ 1867 USA purchase from Russia. In the 1930s, efforts were undertaken to re-introduce the species to former parts of its range. Juveniles were captured in Greenland and transported by ship, trains and boats from Norway to New York to Nunivak Island in the Bering Sea, Alaska. Descendants of these initial founders (<20 individuals) from Nunivak were used in all subsequent translocation throughout Alaska and from Nunivak directly to Wrangel Island (WI) in the Chukotka region of Russia. As a result, all animals at our study sites stem from the same set of founders^45,46^. Muskoxen breeding season commences in August. Gestation averages approximately 240 days and most births occurring in April and May^45^.

### Photogrammetry and Size Assessment

We approximated skeletal growth of muskoxen by using head size dimensionality of 1-, 2-, and 3-yr olds, the ages of most rapid growth^47^, for juvenile cohorts born from 2005–2012 Specifically, we used non-invasive ground photogrammetry to assess linear head dimensions of these readily distinguishable juvenile-aged cohorts^48^. Head size photo images were gathered in March and April (from 2008 through 2013) by on-the-ground approaches to muskoxen along the Alaskan Chukchi realm in and adjacent to National Park Service units involving Bering Land Bridge to Cape Thompson including Cape Krusenstern. We repeated these same methods on WI in March and April, 2014.

### Covariates and Analyses

We used Bayesian path analysis^19^ with covariate data collected from weather stations during the winter, and MODIS vegetation indices during growing seasons, to disentangle potential direct and indirect effects of weather variance on muskoxen and their food resources. Our general conceptual model of hypothesized relationships between covariates of interest (Table 1), and between covariates and muskox head size is given in Fig. 3a. Winter covariates included: mean winter precipitation (November-April), number of extremely cold days (<−23 °C, November-April), and number of days with ROS-enabling conditions from both the previous winter and when an individual was in utero (Fig. 2). Growing season covariates included measures of plant productivity (time integrated normalized difference vegetation index: iNDVI^49^) during the previous growing season and during an individual’s first growing season (Fig. 2). A list of all covariates used in the initial, full models for each age class are given in the Supplementary Material (Table S1). We included covariate data from an individual’s first growing season and from the winter when it was in utero in our models because conditions during early development have been shown to affect muskoxen juvenile growth, and vegetation quality affects maternal milk production^50,51^.

Additional details regarding our Bayesian approach based on Markov chain Monte Carlo (MCMC) simulations to fit path models, as well as parameter estimates for best fit models are in the Supplemental Information and Table S2.

To assess spatial variation in the skeletal growth of muskoxen, we estimated mean age-graded head size of juveniles born from 2005–2012 in the two Alaskan populations and subsequently asked whether similar metrics in the Wrangel Island (Fig. 1) population were within the 90% confidence interval for Alaskan animals.

### Weather Data

We compiled winter weather data for our Alaskan sites (Cape Thompson and Bering Land Bridge) from the closest NOAA-maintained weather stations with data coverage from 2004 to 2013. These were: Deering (Station ID: USW00026643; 66.0689°N, -162.76389°W) for the Bering Land Bridge site, and Kivalina Airport (Station ID: USW00026642; 67.7317°N, -164.5483°W) for the region from Cape Thompson to Krusenstern. Alaskan weather data were accessed from NOAA’s National Center for Environmental Information (NCEI; www.ncdc.noaa.gov/cdo-web). Data used to calculate ROS events on Wrangel Island were from a digital hard-copy offered by the continuously-manned weather station of the Federal Hydro-meteorological Service of Russia in Ushakovskoe (70.9948°N, 178.4845°W) whose weather records date to 1926.

Detection of Arctic ROS events is difficult because weather stations are few, monitoring inconsistent, and ROS relatively rare^52^. Further, snow base and ground temperature modulate characteristics that affect the degree of ground icing that occurs after an ROS event^12,53^. To approximate the annual frequency of ROS events in our study areas, we checked temperature and precipitation records and tallied the number of days when temperatures (T) >0 °C and precipitation occurred, and conditions appropriate for snow to already be on the ground (e. g. previous precipitation had occurred when T < 0 °C). While ground icing can form from repeated melt and freeze events, even when temperatures remain <0 °C, we focus here on conditions that are most likely to result in ground icing.

### Time integrated NDVI

Time integrated NDVI (iNDVI) sums NDVI values of a pixel over an entire growing season, thereby combining information on both peak productivity and growing season length^54^. Our NDVI data covered the ten year period from 2004–2013, were for our two study sites, and obtained from the University of Alaska Geographic Network of Alaska (GINA) (http://gina.alaska.edu/), which receives and publishes MODIS NDVI (eMODIS) datasets from the USGS as 7-day 250 meter resolution composite rasters spanning 42 weeks of each year for the entire state of Alaska (available at: http://gina.alaska.edu/projects/modis-derived-ndvi-metrics). Various annual metrics are then derived for these datasets by GINA to provide several seasonal values of NDVI for each 250 meter pixel, which are then embedded into individual 12-band, 32-bit floating point GeoTiff rasters^49^. Of these metrics, time-integrated NDVI, measured between season onset and season end, were used for analyses.

Our two study areas, Cape Thompson and Bering Land Bridge, were delineated using Google Earth by manually constructing two polygon files. These were then imported into ArcGIS and converted to feature classes. They were then projected to Alaska Albers Equal Area (NAD83)(meters) for further analyses with the NDVI data, which also natively uses this projection system. All geo-processing and spatial analyses were completed using ESRI’s ArcGIS Desktop version 10.2 software^55^.

For each data year, NDVI metric data were exported locally from GINA’s Web Coverage Service (WCS) and clipped to the two study area extents. The time-integrated NDVI metric band was then extracted from each raster along with an additional band indicating pixel value quality (good or bad) using the Composite Band tool in the ArcGIS Spatial Analyst extension. Resulting rasters were then filtered using only good pixel values for each year and study area. Actual time-integrated NDVI values were then inflated by a factor 10^6^ to prevent any truncation of significant digits in the original data prior to analysis. Because we did not have information about individual foraging locations for muskoxen during growing seasons, we assigned each site a yearly mean iNDVI value by averaging the iNDVI values for all pixels at each site in each year.

### Storm surges

To assess storm surges, we coalesced data from NOAA measures of tides and currents (https://tidesandcurrents.noaa.gov/noaatidepredictions/NOAATidesFacade.jsp?Stationid=9491094) for the Red Dog Dock Station (Chart # 16005) which has operated since 2003. The highest observed water level (2.27 m on 2/26/2011) is appreciably greater than mean high water (0.24 m), mean tide level (0.14 m), and mean sea level (0.14 m) across this period.

### Animal Care Assurances

Our work has been performed in accordance with all relevant guidelines and regulations of an animal care institutional committee at Colorado State University and is approved under CSU Protocol ID: 15–6330 A.

## Electronic supplementary material

Supplementary Materials

Supplementary File S1

Supplementary File S2

Supplementary File S3

Supplementary File S4

## References

[1] Putkonen J (2009). Rain on snow: little understood killer in the north. Eos.

[2] Darwin, C. *On the origin of species by means of natural selection*, J. Murray, London (1859).

[3] Post E (2009). Dynamics across the arctic associated with recent climate change. Science.

[4] Rahmstorf S, Coumou D (2011). Increase of extreme events in a warming world. Proc. Nat. Acad. Sci..

[5] Post E (2013). Ecological consequences of sea-ice decline. Science.

[6] Urban MC (2016). Improving the forecast for biodiversity under climate change. Science.

[7] Zhang, K. *et al*. Impacts of large‐scale oscillations on pan‐Arctic terrestrial net primary production. *Geophys. Res. Lett*. **34**, 1029/2007GL031605 (2007).

[8] Billings WD (1987). Constraints to plant growth, reproduction, and establishment in arctic environments. Arct. Alp. Res..

[9] Prowse TD (2006). Climate change effects on hydroecology of arctic freshwater ecosystems. Ambio.

[10] Hansen BB (2013). Climate events synchronize the dynamics of a resident vertebrate community in the High Arctic. Science.

[11] Tyler NJC (2010). Climate, snow, ice, crashes, and declines in populations of reindeer and caribou (*Rangifer tarandus L*.). Ecol. Monogr..

[12] Rennert KJ (2009). Soil thermal and ecological impacts of rain on snow events in the circumpolar Arctic. J. Clim..

[13] Hurley MA (2014). Functional analysis of normalized difference vegetation index curves reveals overwinter mule deer survival is driven by both spring and autumn phenology. Phil. Trans. R. Soc. B.

[14] Ihl C, Klein DR (2001). Habitat and diet selection by muskoxen and reindeer in western Alaska. J. Wildl. Manage..

[15] Larter NC, Nagy JA (2001). Variation between snow conditions at Peary caribou and muskox feeding sites and elsewhere in foraging habitats on Banks Island in the Canadian HighArctic. Arct. Antarc. Alp. Res..

[16] Kumpula J, Colpaert A (2003). Effects of weather and snow conditions on reproduction and survival of semi domesticated reindeer (*R. t. tarandus*). Polar Res..

[17] Van Wijk MT, Williams M, Laundre JA, Shaver GR (2003). Interannual variability of plant phenology in tussock tundra: modelling interactions of plant productivity, plant phenology, snowmelt and soil thaw. Glob. Change Biol..

[18] Bokhorst S (2010). Impacts of extreme winter warming events on plant physiology in a sub‐Arctic heath community. Physiol. Plant..

[19] Shipley, B. *Cause and Correlation in Biology: A User’s Guide to Path Analysis, Structural Equations, and Causal Inference in R*. (Cambridge University Press, Cambridge, 2000).

[20] Köhler M, Marín-Moratalla N, Jordana X, Aanes R (2012). Seasonal bone growth and physiology in endotherms shed light on dinosaur physiology. Nature.

[21] Albon SD, Langvatn R (1992). Plant phenology and the benefits of migration in a temperate ungulate. Oikos.

[22] Peter D, Hanson MA (2004). Maternal constraint of fetal growth and its consequences. Semin. Fetal Neonatal Med..

[23] Muñoz C (2009). Effect of plane of nutrition of 1-and 2-year-old ewes in early and mid-pregnancy on ewe reproduction and offspring performance up to weaning. Animal.

[24] Klein DR (2004). Range-related differences in growth of deer reflected in skeletal ratios. J. Mammal..

[25] Klein DR (1968). The introduction, increase, and crash of reindeer on St. Matthew Island. J. Wildl. Manage..

[26] Lambert, R. E. & Bathgate, J. L. Determination of the plane of nutrition of chamois. *Proc. New Zeal. Ecol. Soc*. 48–56 (1977).

[27] Hewison AJ, Vincent JP, Bideau E, Angibault JM, Putman RJ (1996). Variation in cohort mandible size as an index of roe deer (*Capredus capreolus*) densities and population trends. J. Zool..

[28] Olesen CR, Thing H, Aastrup P (1994). Growth of wild muskoxen under two nutritional regimes in Greenland. Rangifer..

[29] Smith PA, Schaefer JA, Patterson BR (2002). Variation at high latitudes: the geography of body size and cranial morphology of the muskox. Ovibos moschatus. J. Biogeogr..

[30] George JC (2004). Observations on shorefast ice dynamics in Arctic Alaska and the responses of the Iñupiat hunting community. Arctic.

[31] Forchhammer MC, Stenseth NC, Post E, Landvatn R (1998). Dynamics of Norwegian red deer: density-dependence and climatic variation. Proc. R. Soc. B..

[32] Post E (2009). Global population dynamics and hot spots of response to climate change. Bioscience.

[33] Albon SD, Clutton-Brock TH, Guinness FE (1987). Early development and population dynamics in red deer. II. Density-independent effects and cohort variation. J. Anim. Ecol..

[34] Dale BW (2008). Stochastic and compensatory effects limit persistence of variation in body mass of young caribou. J. Mammal..

[35] Metcalfe NB, Monaghan P (2001). Compensation for a bad start: grow now, pay later?. Trends Ecol. Evol..

[36] Berger J (2015). Legacies of past exploitation and climate affect mammalian sexes differently on the roof of the world – The case of wild yaks. Nature – Sci. Rep..

[37] Forbes BC (2016). Sea ice, rain-on-snow and tundra reindeer nomadism in Arctic Russia. Biol. Lett..

[38] Loe LE (2016). Behavioral buffering of extreme weather events in a high‐Arctic herbivore. Ecosphere.

[39] Heide-Jørgensen MP, Richard P, Ramsay M, Akeeagok S (2002). Three recent ice entrapments of Arctic cetaceans in West Greenland and the eastern Canadian HighArctic. NAMMCO Sci. Publ..

[40] Schneider KB, Faro JB (1975). Effects of sea ice on sea otters (*Enhydra lutris*). J. Mammal..

[41] Porsild MO (1918). “savssats”: a crowding of arctic animals at holes in the sea ice. Geog. Rev..

[42] Pearce T, Ford J, Cunsolo-Willox A, Smit B (2015). Inuit traditional ecological knowledge (TEK), subsistence hunting and adaptation to climate change in the Canadian Arctic. Arctic.

[43] Mistry J, Berardi A (2016). Bridging indigenous and scientific knowledge. Science.

[44] Berger, J. US News & World Report http://www.usnews.com/opinion/blogs/world-report/articles/2016-01-08/the-hairy-arctic-mammal-that-thawed-us-russia-relations.

[45] Lent, P. C. *Muskoxen and Their Hunters: A History* (University of Oklahoma Press, 1999).

[46] Berger, J. The science and challenges of conserving large wild mammals in 21st-century American protected areas in *Science, Conservation, and National* Parks (eds Beissinger, S., Ackerly, D., Doremus, H. & Machlis, G. E.) 189–211 (Univ. Chicago Press, 2017).

[47] Peltier TC, Barboza PS (2003). Growth in an Arctic grazer: effects of sex and dietary nitrogen on yearling muskoxen. J. Mammal..

[48] Berger J (2012). Estimation of body‐size traits by photogrammetry in large mammals to inform conservation. Cons. Biol..

[49] Zhu, J. *et al*. MODIS NDVI *Products and Metrics User Manual, Version 1.0* (University of Alaska, 2013).

[50] Clutton-Brock TH, Major M, Albon SD, Guinness FE (1987). Early development and population dynamics in red deer. I. Density-dependent effects on juvenile survival. J. Anim. Ecol..

[51] Forchhammer MC, Clutton-Brock TH, Lindstrom J, Albon SD (2001). Climate and population density induce long-term cohort variation in a northern ungulate. J. Anim. Ecol..

[52] Putkonen J, Roe G (2003). Rain-on-snow events impact soil temperatures and affect ungulate survival. Geophys. Res. Lett..

[53] Conway H, Raymond CF (1999). Snow stability during rain. J. Glac..

[54] Pettorelli N (2011). The Normalized Difference Vegetation Index (NDVI): unforeseen successes in animal ecology. Clim. Res..

[55] ESRI. ArcGIS *Desktop: Release 10.2* (Redlands, CA: Environmental Systems Research Institute, 2013).

