# Supplementary material for: Climate Degradation and Extreme Icing Events Constrain Life in Cold-Adapted Mammals
Source: Sci Rep. 2018 Jan 18;8:1156. doi: 10.1038/s41598-018-19416-9 (PMC5773676; doi:10.1038/s41598-018-19416-9)
Supplement: Supplementary file 1 — Supplementary Materials [file 41598_2018_19416_MOESM1_ESM.pdf]

Supplementary Information for

Climate Degradation and Extreme Icing Events Constrain Life in Cold-Adapted Mammals

Authors: J. Berger[1,2]*, C. Hartway[2,3], A. Gruzdev[4], and M. Johnson[5]

Affiliations:

[1] Fish, Wildlife and Conservation Biology, Colorado State University, Fort Collins, CO, 80523 USA.

[2] Wildlife Conservation Society, Bronx, NY, 10460 USA.

[3] Department of Natural Resources, South Dakota State University, Brookings, SD, 57007 USA.

[4] Wrangel Island World Heritage Site, Pevek, Chukotka Autonomous Zone, RU.

[5] US Forest Service, Tongass National Forest, Juneau, AK, 99801 USA.

*Correspondence to: jberger@wcs.org

This file includes:

Modeling approach

Table S1

Table S2

Other Supplementary Materials for this manuscript include:

R code for calling and running models (Supplementary file S1)

JAGS code for path analysis (full models only) of 1-, 2- and 3-yr olds (Supplementary files S2-S4)

Modeling approach. Our general conceptual model of the hypothesized relationships between covariates of interest, and between covariates and head size for 1-, 2- and 3-yr old muskoxen is given in Fig. 3a in the main text (also see Fig. 2 in text for an understanding of the chronological timing of covariates).

We fit the initial graphical model (Fig 3a) to covariate and head size data from each age class (Table S1) using Bayesian methods based on Markov chain Monte Carlo (MCMC) simulations. For all models we used non-informative normal priors to estimate regression coefficients (mean = 0, s.d. = 100) and uniform priors when estimating variance (range between 0, and 100). We ran an initial burn in of 200,000 iterations with three chains, and generated posterior distributions from a sample of an additional 200,000 iterations. We evaluated convergence using Gelman's diagnostic ($\hat{R}$)[S1]. All simulations were carried out using JAGS[S2], called by rjags[S3] in the R environment[S4]. R code for calling and running models and JAGS code for our initial, full path analysis models for 1-, 2- and 3-yr old muskoxen can be found in the additional Supplementary Materials (Supplementary Files S1 –S4).

We evaluated the fit of our models using a graphical model-fitting approach[S5]. Briefly, the steps of this approach are:

1) Run initial model, examine posterior distributions and credible intervals for all estimated parameters.

2) Drop any "non-significant" covariates or linkages from the model. We defined non-significant covariates or linkages as those for which the 90% credible intervals of estimated regression coefficient included 0. We used methods outlined by Clough (2012)[S6] to estimate the proportion of posterior distributions that did not include 0.

3) Run the pruned model (with all non-significant covariate relationships edited out), and examine relationships between model residuals and all previously dropped covariates to identify potential missing linkages. Re-include back into the model any covariate or linkage that is significantly correlated with model residuals.

4) Run the updated model (with newly found linkages included), re-examine posterior distributions, and repeat steps 2-4 until no further variables, or links between variables, are dropped or added to the graphical model.

All model selection was carried out using un-standardized covariates (results in Table S2). We then re-ran best fit models with standardized covariate values to allow for comparisons of the relative effect size of each covariate on muskoxen head size (results in Fig. 3 in main text).

To assess the goodness-of-fit of final models, we calculated the squared correlation coefficient between observed and predicted values. Because relationships between head size and our chosen covariates appeared to be driven largely by data from the Cape Thompson site (Fig. 4 in text), we also fit our final models to data from each site separately to assess site-specific performance of each model.

References

S1. Gelman, A. & Hill, A. *Data Analysis Using Regression and Multilevel/Hierarchical Models* (Cambridge University Press, 2007).

S2. Plummer, M. JAGS: a program for analysis of Bayesian graphical models using Gibbs sampling. http://mcmc-jags.sourceforge.net (2003).

S3. Plummer, M. Rjags: Bayesian graphical models using MCMC. R package version 4.3. http://mcmc-jags.sourceforge.net (2015).

S4. R Core Team. R: A language and environment for statistical computing. R Foundation for Statistical Computing, Vienna, Austria. https://www.R-project.org (2015).

S5. Grace, J. B. *et al.* Guidelines for a graph-theoretic implementation of structural equation modeling. *Ecosphere* **3,** 1-44 10.1890/ES12-00048.1 (2012).

S6. Clough, Y. A generalized approach to modeling and estimating indirect effects in ecology. *Ecology* **93**, 1809–1815 (2012).

Table S1. A list of all covariates, and the timing during an individual muskox's life when measured, included in the full models for each age class (see Fig. 2 in text for details).

Age class	Timing of covariates	Covariates included in full model
1-yr olds	Winter when in utero	# ROS events, mean monthly winter precipitation, number of days < 23C
	1st growing season	mean time integrated NDVI per site
2-yr olds	Winter when in utero	# ROS events
	1st growing season	mean time integrated NDVI per site
	Previous winter	# ROS events, mean monthly winter precipitation, number of days < 23C
	Previous growing season	mean time integrated NDVI per site
3-yr olds	Winter when in utero	# ROS events
	1st growing season	mean time integrated NDVI per site
	Previous winter	# ROS events, mean monthly winter precipitation, number of days < 23C
	Previous growing season	mean time integrated NDVI per site

Table S2. Parameter estimates for best models fit with un-standardized covariates for 1-yr old, 2-yr old and 3-yr old muskoxen. Covariate codes: mean monthly precipitation during winter when individual is in utero ("mWp"), number of ROS events during winter when individual is in utero ("mROS"), mean site-level iNDVI during the animals first growing season ("NBndvi"), mean monthly precipitation in the previous winter ("Wp"), mean site-level iNDVI during the previous growing season ("ndvi"), number of days during the previous winter when temperature fell below -23°C ("B23c"). Note that for 1-yr olds, the previous winter and the winter when an individual is in utero are the same.

**Model**	**Coefficient**	**Mean**	**SD**	**95% Credible intervals**
1-yr olds	Head size intercept	192.57	6.71	179.42, 205.77
	mWp -> Head size	7.84	4.38	-0.75, 16.44
	NBndvi -> Head size	4.91	2.73	-0.45, 10.26
2-yr olds	Head size intercept	264.41	6.24	252.16, 276.60
	Wp -> Head size	11.49	4.16	3.32, 19.67
	ndvi-> Head size	9.61	2.37	4.94, 14.27
	ndvi intercept	1.05	0.68	-0.30, 2.40
	B23c -> ndvi	-0.05	0.03	-0.10, 0.01
3-yr olds	Head size intercept	341.44	4.14	333.30, 349.55
	mROS -> Head size	-4.58	1.41	-7.35, -1.80
