# Supplementary material for: Climate Degradation and Extreme Icing Events Constrain Life in Cold-Adapted Mammals
Source: Sci Rep. 2018 Jan 18;8:1156. doi: 10.1038/s41598-018-19416-9 (PMC5773676; doi:10.1038/s41598-018-19416-9)
Supplement: Supplementary file 2 — Supplementary File S1 [file 41598_2018_19416_MOESM2_ESM.pdf]

```
library(rjags)

#Input data and run initial model for 1 yo females
load("CandB1_2016Fnew.Rda")
load("CandB1short2.Rda")


### SITE-LEVEL DATA #####
NBndvi <- CandB1short2$NBndvi          #mean site-level iNDVI in individual's 1st growing season
mB23c <- CandB1short2$MLT23            #number of days <23C during gestation
mWp <- CandB1short2$MWpmean            #mean monthly winter precipitation during gestation
mROSnum <- CandB1short2$MROSnum            #number of ROS events during gestation

#transform winter precip. and standardize iNDVI data
mWp_log <- log(mWp+1)
NBndvi_S <- (NBndvi - mean(NBndvi))/sd(NBndvi)

### DATA ON INDIVIDUALS WITHIN SITES ###
HS <- CandB1_2016Fnew$HeadSize                 #Head Size data for 1yo animals
SiteYear <- CandB1_2016Fnew$SiteYear

### CONSTANTS ###
Nsiteyears <- 12
n.ROS <- 180
num_indiv <- c(1, sum(SiteYear==1), sum(SiteYear==2), sum(SiteYear==3), sum(SiteYear==4),
sum(SiteYear==5), sum(SiteYear==6), sum(SiteYear==7), sum(SiteYear==8), sum(SiteYear==9),
sum(SiteYear==10), sum(SiteYear==11), sum(SiteYear==12))

### MODEL INPUTS ###
data.mAfull <- list(n.ROS = n.ROS, mROS = mROSnum, NBndvi = NBndvi_S, mB23c = mB23c, mWp =
mWp_log, num_indiv = num_indiv, Nsiteyears = Nsiteyears, HS = HS)

out_mAfull <- jags.model(file = "Full_1yo_Model_ms.txt", data = data.mAfull, n.chains = 3, n.adapt =
200000)

update(out_mAfull, 200000)
params <- c("B1", "B2", "B3", "B4", "B2.1", "B2.2", "B3.1", "alpha.HS", "alpha.mROS", "alpha.NBndvi")
samps <- coda.samples(out_mAfull, params, n.iter = 200000)
summary(samps)


 ##### Input data and run initial model for 2yo females
load("CandB2_2016F.Rda")
load("CandB2short.Rda")

### SITE-LEVEL DATA #####
ndvi <- CandB2short$ndvi                 #mean site-level iNDVI in previous growing season
B23c <- CandB2short$below23c             #number of days <23C during previous winter
Wp <- CandB2short$Wpmean                 #mean monthly winter precipitation during previous winter
```

```
ROSnum <- CandB2short$ROSnum        #number of ROS events during previous winter
NBndvi <- CandB2short$NBndvi        # remaining covariates codes same as for 1-yr olds
mB23c <- CandB2short$MLT23
mWp <- CandB2short$MWpmean
mROSnum <- CandB2short$MROSnum

#transform winter precip. and standardize iNDVI data
Wp_log <- log(Wp+1)
mWp_log <- log(mWp+1)
ndvi_S <- (ndvi - mean(ndvi))/sd(ndvi)
NBndvi_S <- (NBndvi - mean(NBndvi))/sd(NBndvi)

### DATA ON INDIVIDUALS WITHIN SITES ###
HS <- CandB2_2016F$HeadSize
SiteYear <- CandB2_2016F$SiteYear

### CONSTANTS ###
n.ROS <- 180
Nsiteyears <- 12
num_indiv <- c(1,sum(SiteYear==1), sum(SiteYear==2), sum(SiteYear==3), sum(SiteYear==4),
sum(SiteYear==5), sum(SiteYear==6), sum(SiteYear==7), sum(SiteYear==8), sum(SiteYear==9),
sum(SiteYear==10), sum(SiteYear==11), sum(SiteYear==12))

### MODEL INPUTS ###

data.mAAfull <- list( n.ROS = n.ROS, ROS = ROSnum, ndvi = ndvi_S, B23c = B23c, Wp = Wp_log,
mROS = mROSnum, NBndvi = NBndvi_S, mB23c = mB23c,mWp = mWp_log, num_indiv = num_indiv,
Nsiteyears = Nsiteyears, HS = HS)

out_mAAfull <- jags.model(file = "Full_2yo_Model_ms.txt", data = data.mAAfull, n.chains = 3, n.adapt =
200000)

update(out_mAAfull, 200000)
params <- c("B1","B2", "B3", "B4","B5", "B6", "B1.1", "B1.2", "B2.1", "B2.2", "B3.1", "alpha.HS",
"alpha.ROS", "alpha.mROS", "alpha.ndvi")

samps <- coda.samples(out_mAAfull, params, n.iter = 250000)
summary(samps)


##### Input data and run initial model for 3 yo females
load("CandB3_2016Fnew.Rda")
load("CandB3short3.Rda")

### SITE-LEVEL DATA #####
ndvi <- CandB3short3$ndvi
B23c <- CandB3short3$below23c
Wp <- CandB3short3$Wpmean
ROSnum <- CandB3short3$ROSnum
```

```
NBndvi <- CandB3short3$NBndvi
mWp <- CandB3short3$MWpmean
mB23c <- CandB3short3$MLT23
mROSnum <- CandB3short3$MROSnum

mWp_log <- log(mWp+1)
Wp_log <- log(Wp+1)
ndvi_S <- (ndvi - mean(ndvi))/sd(ndvi)
NBndvi_S <- (NBndvi - mean(NBndvi))/sd(NBndvi)

### DATA ON INDIVIDUALS WITHIN SITES ###
HS <- CandB3_2016Fnew$HeadSize
SiteYear <- CandB3_2016Fnew$SiteYear

### CONSTANTS ###
n.ROS <- 180
Nsiteyears <- 12
num_indiv <- c(1,sum(SiteYear==1), sum(SiteYear==2), sum(SiteYear==3), sum(SiteYear==4),
sum(SiteYear==5),  sum(SiteYear==6), sum(SiteYear==7), sum(SiteYear==8), sum(SiteYear==9),
sum(SiteYear==10), sum(SiteYear==11), sum(SiteYear==12))

### MODEL INPUTS ###
data.mAAfull <- list( n.ROS = n.ROS, ROS = ROSnum, ndvi = ndvi_S, B23c = B23c, Wp = Wp_log,
mROS = mROSnum, NBndvi = NBndvi_S, mB23c = mB23c, mWp = mWp_log, num_indiv = num_indiv,
Nsiteyears = Nsiteyears, HS = HS)

out_mAAfull <- jags.model(file = "Full_3yo_Model_ms.txt", data = data.mAAfull, n.chains = 3, n.adapt =
200000)

update(out_mAAfull, 200000)

params <- c("B1","B2", "B3", "B4","B5", "B6", "B1.1", "B1.2", "B2.1", "B2.2", "B3.1", "alpha.HS",
"alpha.ROS", "alpha.mROS", "alpha.ndvi")

samps <- coda.samples(out_mAAfull, params, n.iter = 200000)
summary(samps)
```
