# Supplementary material for: Climate Degradation and Extreme Icing Events Constrain Life in Cold-Adapted Mammals
Source: Sci Rep. 2018 Jan 18;8:1156. doi: 10.1038/s41598-018-19416-9 (PMC5773676; doi:10.1038/s41598-018-19416-9)
Supplement: Supplementary file 3 — Supplementary File S2 [file 41598_2018_19416_MOESM3_ESM.pdf]

```
#Full Model 1 yos
#Data in from long dataset: HS,
#Data in from short dataset: mROS, mWp, mB23c, NBndvi
#user input: NsiteYears, num_indiv


model {

    #Likelihood
    for (i in 1:Nsiteyears) {

        mROS[i] ~ dbin(mROS.hat[i],n.ROS)
        logit(mROS.hat[i]) <- alpha.mROS + B2.1*mWp[i] + B2.2*mB23c[i]

        NBndvi[i] ~ dnorm(NBndvi.hat[i], tau.NBndvi)
        NBndvi.hat[i] <- alpha.NBndvi + B3.1*mB23c[i]

                for (j in (sum(num_indiv[1:i])):((sum(num_indiv[1:(i+1)]))-1)) {

                HS[j] ~ dnorm(HS.hat[j], tau.HS)
                HS.hat[j] <- alpha.HS + B1*mROS[i] + B2*mWp[i] + B3*mB23c[i] + B4*NBndvi[i]
                }
        }

        #Priors
        B1~ dnorm(0, 0.000001)
        B2 ~ dnorm(0, 0.000001)
        B3 ~ dnorm(0, 0.000001)
        B4 ~ dnorm(0, 0.000001)

        B2.1 ~ dnorm(0, 0.000001)
        B2.2 ~ dnorm(0, 0.000001)
        B3.1 ~ dnorm(0, 0.000001)

        tau.HS <- 1/(sig.HS * sig.HS)
        sig.HS ~ dunif(0,100)

        tau.NBndvi <- 1/(sig.NBndvi * sig.NBndvi)
        sig.NBndvi ~ dunif(0,100)

        alpha.mROS ~ dnorm(0, 0.00001)
        alpha.HS ~ dnorm(0, 0.00001)
        alpha.NBndvi ~ dnorm(0, 0.00001)
        }
```

##We also modeled mROS as an overdispersed poisson distribution; code below. Did not alter model selection results.

```
#mROS[i] ~ dpois(mROS.hat[i])
	#log(mROS.hat[i]) <- log.mROShat[i]
	#log.mROShat[i] <- alpha.mROS + B2.1*mWp[i] + B2.2*mB23c[i] + eps_mROS[i]
	#eps_mROS[i] ~ dnorm(0, tau.eps_mROS)
```

#If using above overdispersed poisson code for mROS, priors must include the terms below:

```
#tau.eps_mROS <- 1/(sig.eps_mROS * sig.eps_mROS)
#sig.eps_mROS ~ dunif(0,10)
```
