# Supplementary material for: Climate Degradation and Extreme Icing Events Constrain Life in Cold-Adapted Mammals
Source: Sci Rep. 2018 Jan 18;8:1156. doi: 10.1038/s41598-018-19416-9 (PMC5773676; doi:10.1038/s41598-018-19416-9)
Supplement: Supplementary file 5 — Supplementary File S4 [file 41598_2018_19416_MOESM5_ESM.pdf]

```
#Full Model 3 yos
#Data in from long dataset: HS,
#Data in from short dataset: ROS, ndvi, Wp, B23c, mWp, mB23c, NBndvi, mROS,
#user input: NsiteYears, num_indiv, n.ROS


model {

    #Likelihood
    for (i in 1:Nsiteyears) {

        ROS[i] ~ dbin(ROS.hat[i],n.ROS)
        logit(ROS.hat[i]) <- alpha.ROS + B1.1*Wp[i] + B1.2*B23c[i]

        mROS[i] ~ dbin(mROS.hat[i],n.ROS)
        logit(mROS.hat[i]) <- alpha.mROS + B2.1*mWp[i] + B2.2*mB23c[i]

        ndvi[i] ~ dnorm(ndvi.hat[i], tau.ndvi)
        ndvi.hat[i] <- alpha.ndvi + B3.1*B23c[i]


          for (j in (sum(num_indiv[1:i])):((sum(num_indiv[1:(i+1)]))-1)) {

                HS[j] ~ dnorm(HS.hat[j], tau.HS)
                HS.hat[j] <- alpha.HS + B1*ROS[i] + B2*ndvi[i] + B3*B23c[i] + B4*Wp[i] +
                B5*NBndvi[i] + B6*mROS[i]
                        }
          }

        #Priors
        B1~ dnorm(0, 0.000001)
        B2 ~ dnorm(0, 0.000001)
        B3 ~ dnorm(0, 0.000001)
        B4 ~ dnorm(0, 0.000001)
        B5 ~ dnorm(0, 0.000001)
        B6 ~ dnorm(0, 0.000001)

        B1.1 ~ dnorm(0, 0.000001)
        B1.2 ~ dnorm(0, 0.000001)
        B2.1 ~ dnorm(0, 0.000001)
        B2.2 ~ dnorm(0, 0.000001)
        B3.1 ~ dnorm(0, 0.000001)

        tau.HS <- 1/(sig.HS * sig.HS)
```

```
	sig.HS ~ dunif(0,100)

	tau.ndvi <- 1/(sig.ndvi * sig.ndvi)
	sig.ndvi ~ dunif(0,100)

	alpha.ROS ~ dnorm(0, 0.00001)
	alpha.mROS ~ dnorm(0, 0.00001)
	alpha.HS ~ dnorm(0, 0.00001)
	alpha.ndvi ~ dnorm(0, 0.00001)

}
```

##We also modeled mROS as an overdispersed poisson distribution; code below. Did not alter model selection results.

```
#ROS[i] ~ dpois(ROS.hat[i])
		#log(ROS.hat[i]) <- log.ROShat[i]
		#log.ROShat[i] <- alpha.ROS + B1.1*Wp[i] + B1.2*B23c[i] + eps_ROS[i]
		#eps_ROS[i] ~ dnorm(0, tau.eps_ROS)

#mROS[i] ~ dpois(mROS.hat[i])
		#log(mROS.hat[i]) <- log.mROShat[i]
		#log.mROShat[i] <- alpha.mROS + B2.1*mWp[i] + B2.2*mB23c[i] + eps_mROS[i]
		#eps_mROS[i] ~ dnorm(0, tau.eps_mROS)


#If using above overdispersed poisson code for mROS, priors must include the terms below:
#tau.eps_ROS <- 1/(sig.eps_ROS * sig.eps_ROS)
#sig.eps_ROS ~ dunif(0,10)

#tau.eps_mROS <- 1/(sig.eps_mROS * sig.eps_mROS)
#sig.eps_mROS ~ dunif(0,10)
```
